# Whole-Cell Vaccine Preparation Through Prussian Blue Nanoparticles-Elicited Immunogenic Cell Death and Loading in Gel Microneedles Patches

**DOI:** 10.3390/gels10120838

**Published:** 2024-12-19

**Authors:** Wenxin Fu, Qianqian Li, Jingyi Sheng, Haoan Wu, Ming Ma, Yu Zhang

**Affiliations:** 1 State Key Laboratory of Digital Medical Engineering, Basic Medicine Research and Innovation Center of Ministry of Education, Southeast University, Nanjing 211102, Chinawuhaoan@seu.edu.cn (H.W.);; 2 Jiangsu Key Laboratory for Biomaterials and Devices, School of Biological Science and Medical Engineering, Southeast University, Nanjing 210096, China

**Keywords:** whole-cell vaccine, lymphoma, immunotherapy, gel microneedle patch, Prussian blue, gelatin methacryloyl, poly(lactic-co-glycolic acid)

## Abstract

Tumor whole-cell vaccines are designed to introduce a wide range of tumor-associated antigens into the body to counteract the immunosuppression caused by tumors. In cases of lymphoma of which the specific antigen is not yet determined, the tumor whole-cell vaccine offers distinct advantages. However, there is still a lack of research on an effective preparation method for the lymphoma whole-cell vaccine. To solve this challenge, we prepared a whole-cell vaccine derived from non-Hodgkin B-cell lymphoma (A20) via the photothermal effect mediated by Prussian blue nanoparticles (PBNPs). The immune activation effect of this vaccine against lymphoma was verified at the cellular level. The PBNPs-treated A20 cells underwent immunogenic cell death (ICD), causing the loss of their ability to form tumors while retaining their ability to trigger an immune response. A20 cells that experienced ICD were further ultrasonically crushed to prepare the A20 whole-cell vaccine with exposed antigens and enhanced immunogenicity. The A20 whole-cell vaccine was able to activate the dendritic cells (DCs) to present antigens to T cells and trigger specific immune responses against lymphoma. Whole-cell vaccines are primarily administered through direct injection, a method that often results in low delivery efficiency and poor patient compliance. Comparatively, the microneedle patch system provides intradermal delivery, offering enhanced lymphatic absorption and improved patient adherence due to its minimally invasive approach. Thus, we developed a porous microneedle patch system for whole-cell vaccine delivery using Gelatin Methacryloyl (GelMA) hydrogel and n-arm-poly(lactic-co-glycolic acid) (n-arm-PLGA). This whole-cell vaccine combined with porous gel microneedle patch delivery system has the potential to become a simple immunotherapy method with controllable production and represents a promising new direction for the treatment of lymphoma.

## 1. Introduction

The lymphatic system comprises lymph nodes, tonsils, thymus, spleen, and bone marrow that are distributed throughout the body and interact with the mononuclear macrophage and blood systems. Lymphoma is a diverse lymphocytic malignancy that can manifest in lymphoid tissue and/or extranodal sites throughout the body and lead to various symptoms [1,2,3,4,5,6]. The occurrence and progression of lymphoma are tightly correlated with the immune function of host. Specifically, the abnormal proliferation of tumor cells in lymphoid organs causes damage to lymphoid tissues, and further impairs the immune system function, which in return weakens the anti-tumor immune response and fosters tumor proliferation [7]. According to the World Health Organization and its Global Burden of Disease Project, the incidence rate and range of malignant lymphoma are increasing worldwide, posing a significant threat to human life and health [8,9,10,11]. Non-Hodgkin lymphoma (NHL) represents over 80% of malignant lymphoma cases and diffuse large B-cell lymphoma (DLBCL) is the most common subtype that accounts for approximately 30% to 40% of NHL cases [12,13,14,15,16]. DLBCL exhibits high heterogeneity in clinical manifestations, molecular genetics, and immunophenotypes. The poor prognosis and high recurrence rate of DLBCL cause a significant challenge in its clinical treatment [17,18,19,20].

In recent years, immunotherapy has been introduced to treat DLBCL. Immunotherapy activates patient’s own immune system by enhancing, guiding, or restoring the body’s natural defense capacity to combat cancer. Targeting tumor with adaptive immune system offers specificity and holds promise in avoiding tissue damage caused by conventional therapies such as chemotherapy and radiotherapy [21]. Cancer vaccine has been recognized as a reliable and effective form of tumor immunotherapy. Cancer vaccines are categorized into cell vaccines (tumor or immune cells), protein/peptide vaccines, and gene (DNA, RNA, and virus) vaccines based on their form or content. Introducing a specific cancer treatment vaccine to patients can overcome the immunosuppression state caused by tumors, enhance tumor immunogenicity, activate the patient’s immune system, induce cellular and humoral immune responses, and ultimately control or eliminate the tumor [22,23]. However, NHL lacks specific antigens which the vast majority of tumor vaccines work on [24]. While cell vaccine is abundant in antigen epitopes and MHC class I and II-restricted antigens, which can prompt a comprehensive and effective anti-tumor response and the generation of long-term memory T cells [25,26]. More importantly, whole-cell vaccines utilize the entire tumor cell as an immunogenic source do not require prospective identification of tumor antigens, and can target multiple antigens concurrently, thus offering unique advantages to lymphoma treatment [27].

Immunogenic cell death (ICD) represents a distinct form of regulatory cell death [28]. Specifically, stressed and dead mammalian cells release various bioactive molecules, such as small metabolites (e.g., adenine nucleoside triphosphate (ATP)), nucleic acids, proteins (e.g., cytokines and damage-associated molecular patterns), and lipids (e.g., oxidized cardiolipin). These molecules interact with the immune system to determine the immunogenicity of cell stress and death [29,30], disrupt immune tolerance, and initiate an anti-tumor immune response [31]. Leveraging the effectiveness of ICD to kickstart anti-tumor immunity against a variety of antigens released by dying tumor cells holds promise as a potent and adaptable strategy for personalized cancer immunotherapy.

The traditional method of preparing a tumor whole-cell vaccine involves using physical (magnetothermal, photothermal), chemical, or biological methods (such as ultraviolet radiation, heating, neuraminidase, etc.) to induce ICD in autologous or allogeneic tumor cells. The resulting vaccine retains its ability to trigger an immune response without causing tumor progression. When administered to the patient, it can elicit a comprehensive immune response targeting the tumor [32,33,34,35,36]. Various studies have unveiled the potential of whole-cell vaccines in treating lymphoma, presenting a new prospect for DLBCL immunotherapy [37,38,39,40,41]. However, an effective method for preparing a whole-cell vaccine for DLBCL still remains lacking.

Prussian blue nanoparticles (PBNPs) have been found to convert near-infrared laser into thermal energy and raising the system’s temperature, their photothermal performance has been observed in various sizes and shapes [42]. In a study by Fu et al. [43], PBNPs were used as a photothermal conversion agent to treat tumors. It was observed that when PBNPs were exposed to an 808 nm laser for 3 min, the system temperature increased to 43 °C, demonstrating the efficient photothermal conversion capability of PBNPs. Previous research from our group’s key research and development project indicated that the presence of PBNPs in the culture of lymphoma cell lines might enhance the cancer stemness of lymphoma cells [44], consequently has the potential to boost the ICD of the lymphoma cells.

In targeted drug delivery for lymphoma, the method of administration plays a crucial role in influencing lymphatic absorption. When drugs are administered interstitially, nanocarriers accumulate in the tissue interstitium and are more likely to enter lymphatic vessels due to the gaps present between endothelial cells [45,46]. Intradermal administration is particularly effective, showcasing the highest lymphatic absorption efficiency owing to increased interstitial pressure and enhanced lymphatic flow velocity [47]. Microneedles have emerged as a minimally invasive and painless delivery method that has garnered significant attention from researchers [48]. Their versatility is enhanced by various manufacturing options, and they are user-friendly, requiring no specialized training. Microneedle systems facilitate continuous drug release and offer flexible dosing schedules [49,50]. For DLBCL immunotherapy, microneedles present a promising avenue by streamlining vaccination processes, improving patient compliance, and enabling precise intradermal delivery. Currently, the delivery of cancer vaccines is one of the most extensively explored applications of microneedles [51].

The development of whole-cell tumor vaccines is currently constrained by challenges related to preparation and delivery methods. Many processing techniques can diminish the immunogenicity of tumor cells, while inefficient delivery methods often lead to the wasting of valuable autologous vaccines. This study aims to advance the creation of whole-cell vaccines specifically for B-cell NHL and to develop microneedle patches for enhanced delivery, in order to provide new directions for immunotherapy of DLBCL. In this study, we propose a method for preparing a whole-cell vaccine for malignant lymphoma utilizing the photothermal effect mediated by PBNPs. The ICD in non-Hodgkin’s B-cell lymphoma cells (A20) was induced by the photothermal effect of PBNPs upon near-infrared laser irradiation. The optimal laser power density (LPD) for the photothermal treatment was determined by detecting ICD markers high mobility group protein B1 (HMGB1), calreticulin (CRT), and ATP, and the ICD effect achieved by PBNP photothermal treatment and conventional hydrothermal treatment was compared. To prepare the whole-cell vaccine, the treated A20 then experienced ultrasonic fragmentation to expose antigens to further enhance its immune activation effect. The immune cell activation effect of the whole-cell vaccine was characterized. The study confirmed that the A20 whole-cell vaccine underwent ICD and could activate immune cells. This whole-cell vaccine has the potential to become a simple immunotherapy method with controllable production and represents a promising new approach for DLBCL treatment. We also designed porous poly(lactic-co-glycolic acid) (PLGA) microneedles integrated with Gelatin Methacryloyl (GelMA) light-cured hydrogel, which featured a porous structure that can load drugs and had mechanical strength to ensure puncture. This innovative delivery system enabled intradermal vaccination with a sustained release from the microneedles, aimed at improving vaccine utilization and enhancing patient compliance. This whole-cell vaccine with gel microneedle system has the potential to become a simple immunotherapy method with controllable production and represents a promising new approach for DLBCL treatment.

## 2. Results and Discussion

### 2.1. ICD Induced by Photothermal Effect Mediated by PBNPs In Vitro

**Figure 1:**
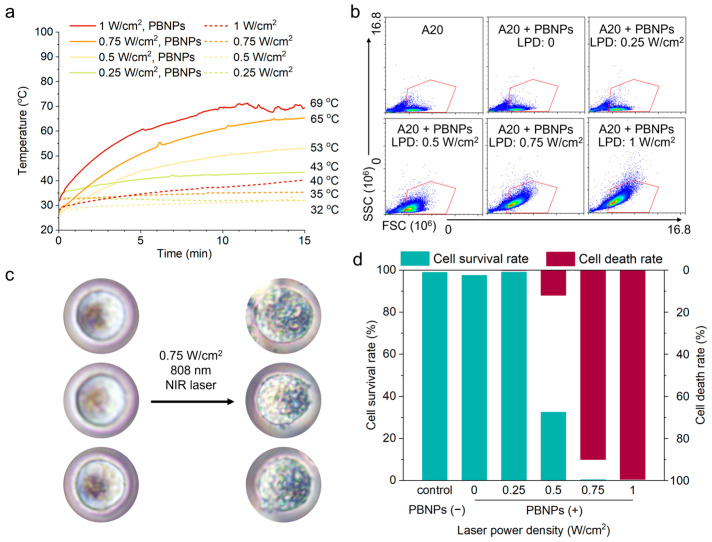
(**a**) Temperature changes and final temperature of A20 photothermal system with or without Prussian blue nanoparticles (PBNPs) under different laser power densities (LPDs). (**b**) Flow cytometric scatter diagrams of A20 cells after different treatments. (**c**) Changes in the optical microstructure of cells before and after photothermal treatment (808 nm laser, 0.75 W/cm^2^) in A20. (**d**) The survival and death rates of A20 cells following photothermal treatment with different LPDs.

The A20 photothermal system’s temperature change mediated by 0.1 mg/mL of PBNPs (square, particle size 175.8 nm, with surface modified poly(N-vinylpyrrolidone) (PVP, K30)) was monitored using an industrial thermometer (Figure 1a). A20 PBNPs cell suspension was exposed to an 808 nm laser with LPD of 0.25 W/cm^2^, 0.50 W/cm^2^, 0.75 W/cm^2^, or 1.00 W/cm^2^ for 15 min using a cell phototoxicity irradiator. The control group consisted of A20 cell suspension without PBNPs. The results revealed that the initial and final temperatures of the control group without PBNPs did not change by more than 10 °C at each power intensity. However, at a LPD of 1.00 W/cm^2^, the final temperature of the control group exceeded 40 °C, while the final temperature of each control group did not exceed 35 °C. In the presence of PBNPs, each group experienced a gradual temperature rise, with the final temperature reaching 43 °C at 0.25 W/cm^2^ LPD, 53 °C at 0.50 W/cm^2^ LPD, 65 °C at 0.75 W/cm^2^ LPD, and 69 °C at 1.00 W/cm^2^ LPD. The results of the heating characterization demonstrate that PBNPs efficiently heat the A20 cell system. Furthermore, it was observed that the heating rate increased as the LPD was under 1 W/cm^2^.

In order to determine the laser power treatment density of ICD in A20, it is necessary to assess the impact of photothermal-induced A20 cell death and eliminate laser power treatment densities that do not meet cytotoxicity requirements. Utilizing the cell death Staining Kit (calcein AM & PI) in conjunction with flow cytometry, cell death was evaluated following exposure to 808 nm LPDs of 0, 0.25 W/cm^2^, 0.50 W/cm^2^, 0.75 W/cm^2^, and 1.00 W/cm^2^ for 15 min, with untreated A20 cells serving as the control group. Figure 1b illustrates the scatter diagram of cells detected by flow cytometry, where the horizontal axis represents forward scattered light (FSC) denoting cell size, and the vertical axis represents laterally scattered light (SSC) indicating particle complexity in cells. The results revealed a significant increase in SSC, signifying heightened particle complexity in A20 cells with escalating photothermal power, consistent with microscopic observations. Before and after treatment with an 808 nm LPD of 0.75 W/cm^2^ for 15 min, the morphology of A20 cells under the optical microscope changed significantly (Figure 1c). The cytoplasm of A20 cells exhibited uniform transparency, distinct cytoplasmic nuclei, and coherent, complete cell edges before heating, while the cytoplasm of A20 cells had a complex optical structure with small granular blocks and dark color after heating. Meanwhile, after heating treatment, the distinction between the cytoplasm and the nucleus was obscured and the cell edge became slightly rough. The structural changes indicated the significant impact of higher-power photothermal treatment on A20 cells. Figure 1d presents the survival and death rates of A20 cells under photothermal treatment with varying LPD. The results indicated that PBNPs alone did not influence the survival rate of A20 cells. Notably, at a LPD of 0.25 W/cm^2^, the cell survival rate post-photothermal treatment reached 99%, demonstrating minimal cell death. Conversely, at LPDs of 0.50 W/cm^2^, 0.75 W/cm^2^, and 1.00 W/cm^2^, a notable decline in survival rates was observed, with over 99% cell death at 0.75 W/cm^2^ and 1.00 W/cm^2^, indicating a pronounced photothermal killing effect. Based on the analysis, subsequent research on the ICD effect resulting from photothermal treatment under various LPD of A20 will exclude 0.25 W/cm^2^ and focus on LPD of 0.50 W/cm^2^, 0.75 W/cm^2^, and 1.00 W/cm^2^.

**Figure 2:**
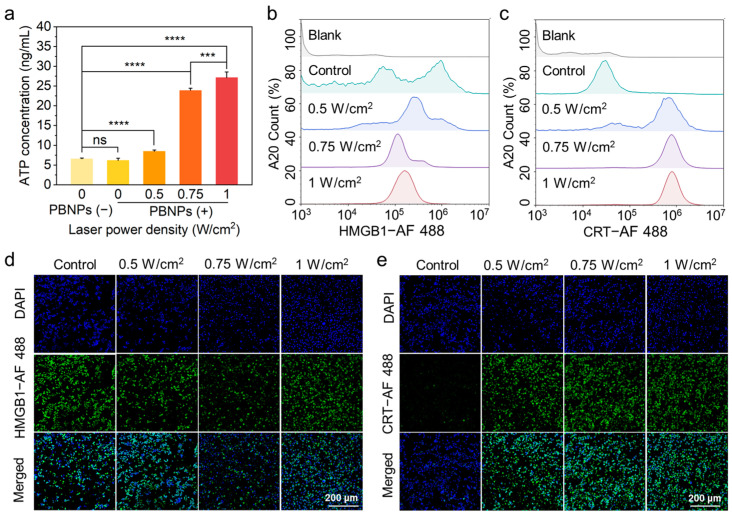
(**a**) ATP content secreted by A20 cells after photothermal treatment with different LPDs. (**b**) Flow cytometry characterization results of high mobility group protein B1 (HMGB1) content in A20 cytoplasm after photothermal treatment with different LPDs. (**c**) Flow cytometry characterization results of calreticulin (CRT) content in A20 cell membrane after photothermal treatment with different LPDs. (**d**) Confocal fluorescence images of HMGB1 content in A20 cytoplasm treated with different LPDs (20×). (**e**) Confocal fluorescence images of CRT content in A20 cell membrane treated with different LPDs (20×). *** *p*  <  0.001, **** *p*  <  0.0001, ns: not significant.

A20 cells can demonstrate enhanced immune activation when ICD occurs, potentially leading to more effective DLBCL treatment. Common ICD indicators of ICD include ATP, HMGB1, and CRT of A20 after heating were characterized to evaluate ICD induction efficiency of different LPDs. ATP levels increase in the extracellular environment during ICD. A general adenosine triphosphate ELISA kit was used to detect ATP secretion in the extracellular supernatant of cells treated with 808 nm LPDs of 0, 0.50 W/cm^2^, 0.75 W/cm^2^, and 1.00 W/cm^2^ for 15 min. The control group consisted of untreated A20 cells. the ATP content in the supernatant after photothermal treatment with different LPDs is shown in Figure 2a. The results indicate that the presence of PBNPs alone did not affect ATP secretion in the supernatant. Compared to the 0.50 W/cm^2^ LPD, ATP secretion in the supernatant significantly increased at LPDs of 0.75 W/cm^2^ and 1.00 W/cm^2^.

During ICD, the content of HMGB1 in cells decreases, the expression of CRT on cell membrane surface increases. The contents were detected using immunofluorescence, flow cytometry, and confocal microscopy after treatment with 808 nm LPDs of 0.50 W/cm^2^, 0.75 W/cm^2^, and 1.00 W/cm^2^ for 15 min, followed by a 4-h incubation in a cell incubator. The control group consisted of untreated A20 cells. The flow cytometry results (Figure 2b,c) showed the difference in HMGB1 content in the cytoplasm and CRT on the membrane surface of A20 cells after photothermal treatment at different LPDs detected by flow cytometry. Additionally, the results of confocal microscopy observation were inconsistent with flow cytometry results. The confocal images showed the differences in the contents of HMGB1 in the cytoplasm and CRT on the membrane surface of A20 cells after photothermal treatment with different LPDs (Figure 2d,e). The HMGB1 levels in A20 cells treated with LPDs of 0.50 W/cm^2^, 0.75 W/cm^2^, and 1.00 W/cm^2^ were significantly lower than control group, with the lowest level observed in the 0.75 W/cm^2^ treatment group. Similarly, compared with control group, the cell membrane surface CRT of each group increased significantly, with the 0.75 W/cm^2^ and 1.00 W/cm^2^ treatment groups showing the highest content of cell membrane surface CRT without significant difference.

In conclusion, based on the expression results of three ICD indicators, ATP secretion in extracellular supernatant, HMGB1 content in the cytoplasm, and CRT exposure on the cell membrane surface, the optimal LPD for the preparation of A20 whole-cell vaccine was determined to be 0.75 W/cm^2^ laser photothermal LPD.

To assess whether the photothermal effect mediated by PBNPs can more effectively induce A20 ICD than conventional water bath heat treatment, we characterized the three ICD indicators of A20 extracellular supernatant ATP secretion, cytoplasmic HMGB1 content, and CRT exposure on cell membrane surface under different heat treatment methods, following the same principle as described before.

**Figure 3:**
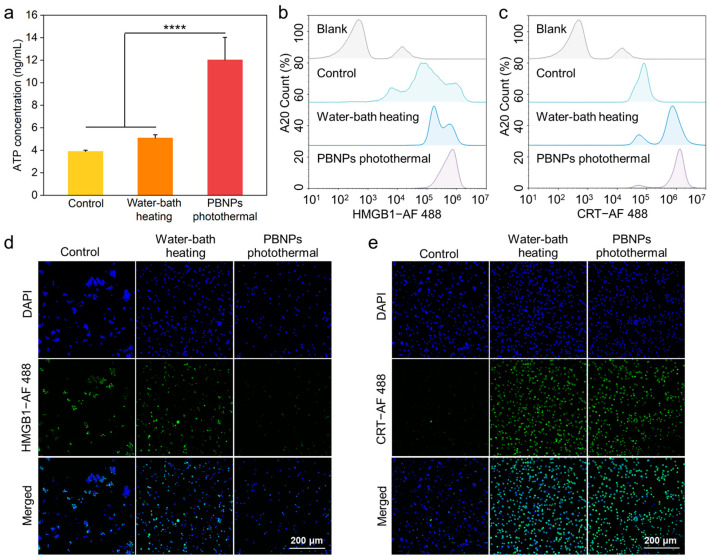
(**a**) ATP content secreted by A20 cells after different heat treatments. (**b**) Flow cytometry characterization results of HMGB1 in A20 cytoplasm after different heat treatments. (**c**) Flow cytometry characterization results of CRT expression on A20 cell membrane after different heat treatments. (**d**) Confocal fluorescence images of HMGB1 content in A20 cytoplasm after different heat treatments (20×). (**e**) Confocal fluorescence images of CRT expression on A20 cell membrane after different heat treatments (20×). **** *p*  <  0.0001.

Figure 3a showed the ATP content in the supernatant after different heat treatments (water bath heating and PBNPs mediated photothermal). The results revealed that the ATP secretion of A20 under photothermal treatment mediated by PBNPs was significantly higher than that of A20 treated with water bath heating and A20 in the control group. In Figure 3b,d, the variations in HMGB1 content in A20 cells following different heat treatments were illustrated using flow cytometry and confocal microscopy. The PBNPs-mediated photothermal effect treatment group exhibited the lowest HMGB1 content in the cytoplasm of A20 cells compared to the control group and the water bath heating treatment group, suggesting a more effective triggering of A20 ICD. Figure 3c,e depicted the differences in CRT expression on the surface of A20 cells following various heat treatments, as detected by flow cytometry and confocal microscopy. The PBNP-mediated photothermal effect treatment group showed the highest CRT expression on the cell membrane surface compared to the control group and the water bath heating treatment group, indicating a more effective triggering of A20 ICD.

Based on the analysis of ATP secretion in the extracellular supernatant, HMGB1 content in the cytoplasm, and CRT exposure on the cell membrane surface, PBNP-mediated photothermal treatment can trigger more effective ICD of A20 than water bath treatment.

### 2.2. Preparation and Characterization of A20 Whole-Cell Vaccine

**Figure 4:**
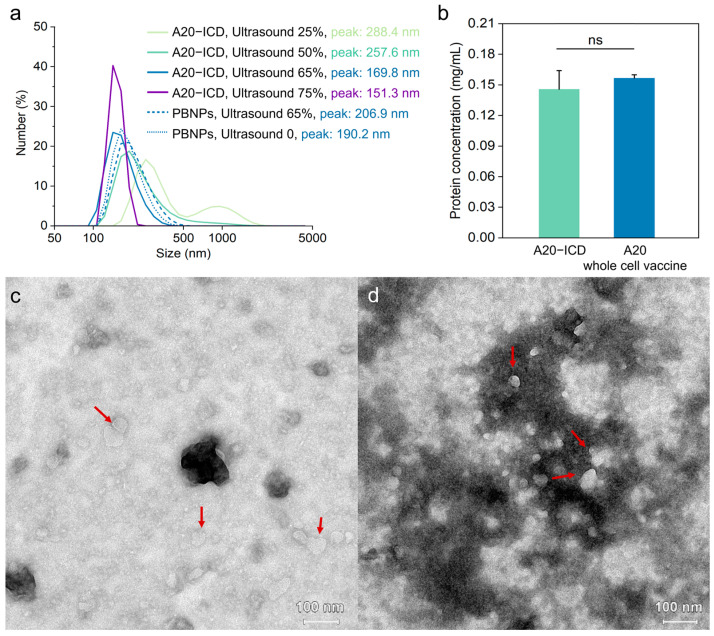
(**a**) Particle size distribution of A20 that underwent ICD (A20–ICD) ultrasonic fragmentation products under different ultrasound powers. (**b**) Protein content of A20–ICD and its ultrasonic fragmentation products. (**c**) transmission electron microscope (TEM) image (100×) of A20–ICD lysate products (red arrow indicates) within 1 h after ultrasonic fragmentation. (**d**) TEM image (100×) of A20–ICD lysate products (red arrow indicates) 10 days after ultrasonic fragmentation. ns: not significant.

In order to enhance the immunogenicity of A20 that underwent ICD (A20–ICD), A20–ICD was treated by an ultrasonic fragmentation method to expose more antigens. After crushing, the particle size of A20 cells was measured using a particle size potential analyzer (Figure 4a), and the morphology and particle size of the broken products were observed using a transmission electron microscope (TEM) (Figure 4c). Generally, particles with a size of less than 200 nm have a better effect on lymph node targeting and dendritic cells (DCs) activation [52,53,54,55]. The ultrasonic cell crusher was used to crush cells at 25%, 50%, 65%, and 75% (100%: 650 W) power, and the control group was 0.1 mg/mL PBNPs. Figure 4a showed the particle size distribution and peak position data of cell fragmentation products measured by the particle size potential analyzer. When the ultrasonic power is 25% and 50%, the comprehensive particle size range of fragmentation products was significantly larger than 200 nm. For the ultrasonic power of 65% and 75%, the particle size range of fragmentation products is less than 200 nm. However, the temperature of ultrasonicated A20 suspension remained high even under ice bath conditions at 75% ultrasonic power. Based on the measurement results of the particle size potential analyzer, 65% power was determined to be the appropriate ultrasonic breaking power for cells. The ultrasonic test of PBNPs control group solution was carried out at 65% ultrasonic power. As shown in Figure 4a, the particle size of PBNPs did not change significantly before and after ultrasound, which suggests that the existence of PBNPs did not affect the measurement of the particle size of the system.

In order to observe and statistically analyze the size and morphology of the whole-cell vaccine more accurately, eliminate measurement errors in hydrodynamic dimensions, and test the stability of the prepared whole-cell vaccine, the morphology of whole-cell vaccine was observed using TEM. The samples of cell fragmentation products were either tested within 1 h (Figure 4c) after crushing or after 10 days (Figure 4d). As indicated by arrows, the broken cell products contained numerous vesicles with membrane structures, and their morphology and particle size were relatively random and uneven. Most particles were rough-edged, nearly circular, with a particle size mostly below 50 nm. Some particles had slightly larger sizes, but overall, the particle size was less than 100 nm and did not significantly change after 10 days of storage. Based on the particle size potential analyzer and the electrical transmission observations, it was determined that 65% (422.5 W) ultrasonic power would be used to crush the cells.

To further confirm the impact of the fragmentation step on the A20 whole-cell vaccine, it is important to standardize the vaccine’s content and employ the BCA protein content detection method for normalization. Figure 4b showed that there was no significant difference in protein content between A20–ICD and A20 whole-cell vaccine (ultrasound-fragmented A20–ICD). This indicates that the ultrasonic fragmentation step does not affect the content of the A20 whole-cell vaccine and it is not necessary to normalize the concentration after sonication process.

### 2.3. In Vitro DCs and T Cells Activation Effect of A20 Whole-Cell Vaccine

**Figure 5:**
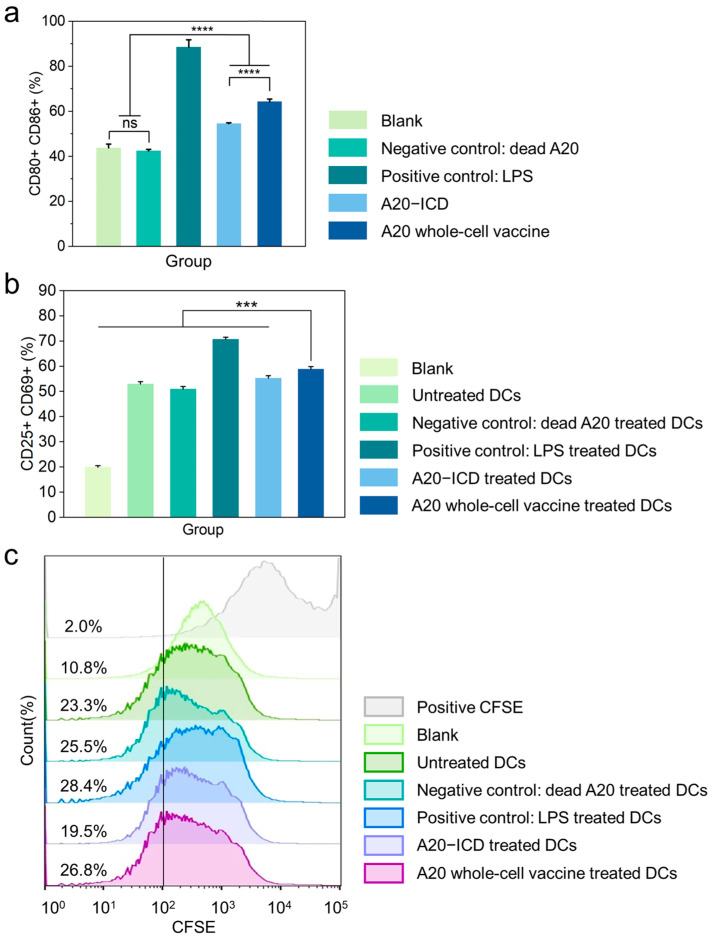
(**a**) Activation rate of bone marrow dendritic cells (BMDCs) stimulated with different treatments. (**b**) Activation rate of T cells stimulated with different treatments. (**c**) The proliferative effect of T cells stimulated with different treatments. *** *p*  <  0.001, **** *p*  <  0.0001, ns: not significant.

The activation of DCs is a critical step in the presentation of antigens to T cells and the initiation of the immune response pathway. In our study, A20–ICD and A20 whole-cell vaccine were employed as stimuli to activate bone marrow dendritic cells (BMDCs). Dead A20 treated with 75% ethanol was utilized as the negative control, while lipopolysaccharide (LPS) served as the positive control. The activation index of BMDCs was assessed using flow cytometry, and BMDC activation was determined by evaluating the expression rate of CD11c, CD80, and CD86. In the flow cytometry results, a single cell population was selected, dead cells were excluded, positive expression of CD11c was used as the clustering index of BMDCs, and the double positive expression of CD80 and CD86 was considered as the marker of BMDCs activation. The results (Figure 5a) revealed that the average activation rate of DCs without stimulation (blank group), DCs stimulated by 75% ethanol-treated A20 (negative control group), DCs stimulated by LPS (positive control group), DCs stimulated by A20–ICD, and DCs stimulated by A20 whole-cell vaccine was 43.8%, 42.4%, 88.5%, 54.5%, and 64.3%, respectively. It was noted that the activation of the blank group and negative control group was influenced by the added stimulation factors during the culture process, resulting in an increase. There was no significant difference in the activation rate of DCs between the blank group and the negative control group. Notably, A20–ICD and A20 whole-cell vaccine demonstrated significantly higher activation rates of DCs compared to the blank and negative control groups. Moreover, A20 whole-cell vaccine exhibited a significantly higher activation rate of DCs than the A20–ICD group. In summary, A20–ICD and A20 whole-cell vaccine were all capable of activating DCs and A20 whole-cell vaccine demonstrated more effective activation of DCs than A20–ICD, suggesting the whole-cell vaccine has the potential to stimulate the body’s immune pathway.

DCs interact with specific receptors on the surface of T cells, providing activation signals that promote T cell differentiation, proliferation, and the generation of effector T cells, including cytotoxic T cells and helper T cells. In this study, various groups of active DCs were co-cultured with primary mouse spleen T cells to induce the activation of the T cells. The untreated DCs group (blank DCs) and the DCs stimulated with 75% ethanol-treated A20 served as the negative control group of T cells, and the DCs stimulated with LPS served as the positive control group of T cells. The activation rate of T cells was determined using flow cytometry, and T cell activation was assessed based on the expression rate of CD3, CD25, and CD69. In the flow cytometry results, a single cell population was selected, dead cells were excluded, positive expression of CD3 was used as the clustering index of T cells, and the double positive expression of CD25 and CD69 was used as the marker of T cell activation. The results (Figure 5b) revealed that the average activation rate of T cells without stimulation (blank group), T cells stimulated by untreated DCs (negative control group), T cells stimulated by DCs with 75% ethanol-treated A20 (negative control group), T cells stimulated by DCs with LPS (positive control group), T cells stimulated by DCs with A20–ICD, and T cells stimulated by DCs with A20 whole-cell vaccine was 21.2%, 40.4%, 26.2%, 59.5%, 41.9%, and 42.7%, respectively. The findings indicated that A20–ICD and A20 whole-cell vaccine both exhibited significantly higher T-cell activation rates compared to the blank group and the negative control group. Notably, the A20 whole-cell vaccine demonstrated significantly higher T-cell activation rate. In conclusion, A20–ICD and A20 whole-cell vaccine both possess the capability to stimulate DCs and present antigens to further activate T cells, with A20 whole-cell vaccine showing the more potent T cell activation effect.

T cells undergo proliferation following stimulation and activation. The CFSE dye fluorescence intensity gradually decreases by half with each cell division, therefore, the distribution of fluorescence intensity represents the extent of T cell proliferation (Figure 5c). The positive CFSE group, which did not receive any treatment, exhibited a CFSE fluorescence intensity higher than 10^2^, serving as the reference value for T cells. In comparison, the CFSE fluorescence intensity of the other groups significantly decreased, indicating cell division and proliferation. Among them, the unstimulated T-cell group displayed a single narrow peak of fluorescence distribution and the highest average intensity of CFSE fluorescence, suggesting the lowest degree of proliferation. T cells stimulated by untreated DCs showed sub-concentrated and sub-strong CFSE fluorescence signals, indicating a relatively lower degree of proliferation. The negative control T cells stimulated by DCs with 75% ethanol-treated A20 and the T cells stimulated by DCs with A20–ICD exhibited similar, relatively dispersed, and low-intensity CFSE fluorescence signals, indicating active cell proliferation. The positive control T cells stimulated by DCs with LPS and the T cells stimulated by DCs with A20 whole-cell vaccine showed the most dispersed CFSE fluorescence signals with the lowest average intensity, demonstrating extremely active cell proliferation. In conclusion, A20–ICD and A20 whole-cell vaccine all have the capacity to activate T-cell proliferation while the A20 whole-cell vaccine has the most potent effect on T-cell activation and proliferation.

### 2.4. Loading and Release of Porous Microneedles for Whole-Cell Vaccines

**Figure 6:**
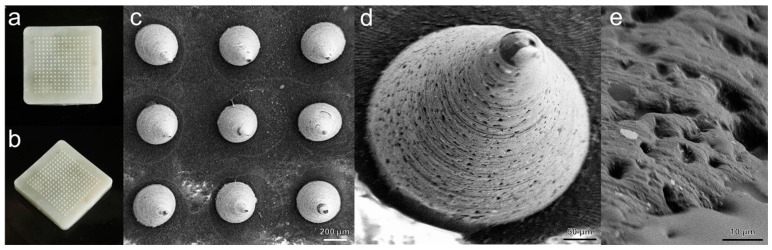
Poly(lactic-co-glycolic acid) (PLGA) porous microneedle patch (**a**) Top view photo. (**b**) Side view photo. (**c**) Surface mounted microneedle array scanning electron microscope (SEM) image (100×). (**d**) SEM image of microneedle body (500×). (**e**) SEM image of surface pore structure of microneedle body (5 k×).

PLGA porous microneedles are formed through thermal crosslinking and acid etching, achieving a stable 100% needle yield. The micro needle patch structure is complete, the base is flat, the needle body is uniform, and the needle tip is sharp. Further observation of the prepared PLGA porous microneedles under scanning electron microscope (SEM) (Figure 6) reveals that the microneedle body is a relatively standard conical shape, with uniformly distributed pores with a diameter range of 5–10 μm on its surface, the porosity of microneedles is 12.54%. This proves the successful construction of the porous microneedle body pore structure. By using the Instron series material testing machine to compress the microneedle patch system, the microneedles broke within 500 μm with a fracture force of 0.23 N/needle, which theoretically provides sufficient mechanical strength to puncture the human epidermis (Figure S4) [56].

**Figure 7:**
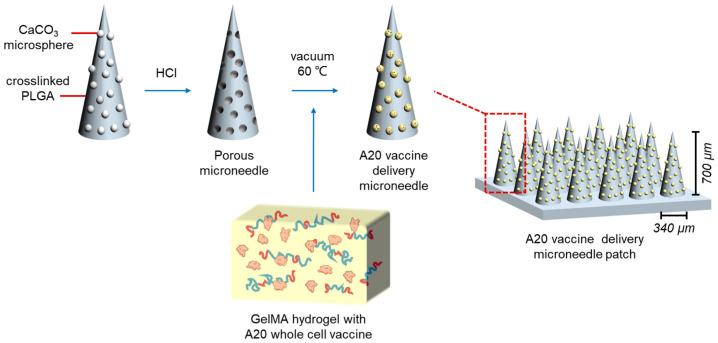
Construction principle of PLGA porous microneedles with Gelatin Methacryloyl (GelMA) hydrogel.

**Figure 8:**
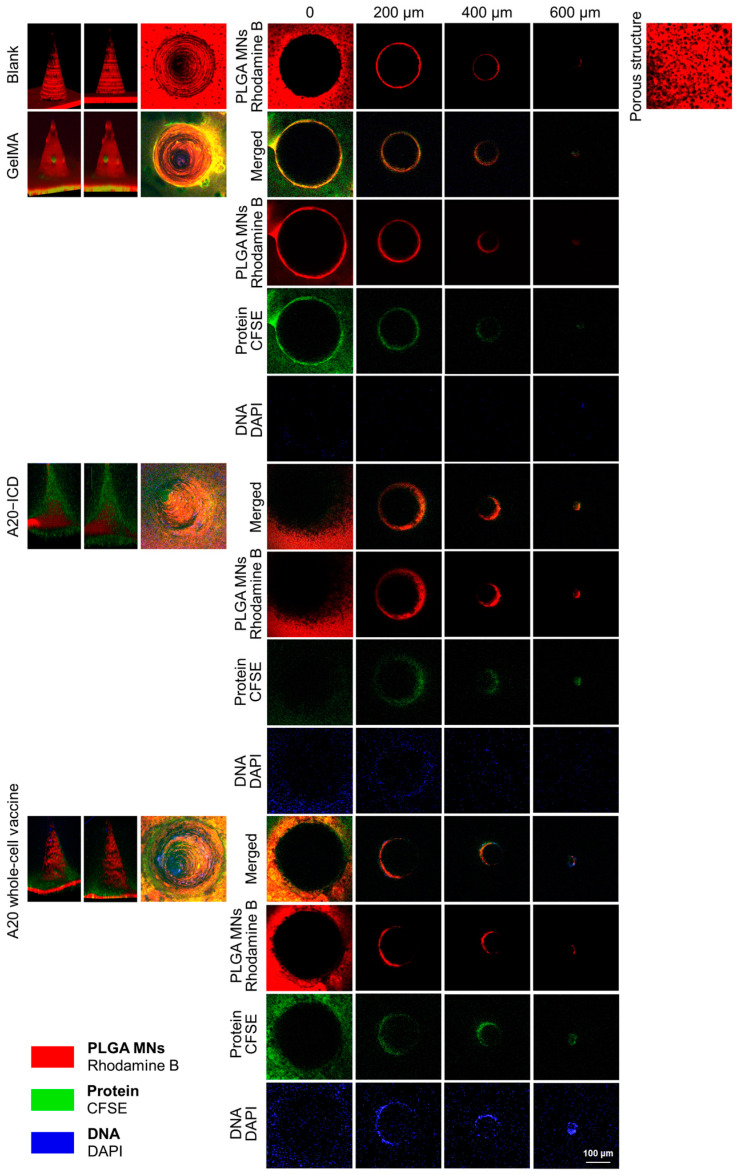
Loading of porous microneedle system for A20 whole-cell vaccine (10×, scale bar: 100 μm, scanning of confocal layer from 0 to 600 μm indicating microneedle bottom to tip).

In the prepared PLGA porous microneedles, GelMA was used as a carrier to carry A20–ICD and A20 whole-cell vaccine (Figure 7, Figures S5 and S6). The PLGA microneedle body was labeled with Rhodamine B, Protein and nucleic acid were labeled with CFSE and DAPI staining solutions, respectively. The fluorescence results of the two showed the loading status of A20 whole-cell vaccine. As shown in Figure 8, the scanning results of the confocal fluorescence layer porous microneedles without-loading showed that the morphology of the prepared microneedles was uniform, and the fluorescence intensity of Rhodamine B in each layer was basically consistent and standard circular. The needle body was a relatively standard conical shape, with a needle length of about 700 μm. The surface of the microneedles showed obvious pore structures, which further confirmed the previous observation of the morphology and structure of porous microneedles. It can be considered that PLGA porous microneedles were successfully prepared. The porous microneedle equipped with GelMA displays CFSE fluorescence, which was wrapped around the outer layer of Rhodamine B fluorescence. The CFSE fluorescence label was dark or invisible near the needle tip, while the DAPI fluorescence label was not displayed in the entire structure of the needle body. The fluorescence characteristics of the needle body labeled with Rhodamine B using A20–ICD porous microneedles are consistent with those of the blank group microneedles. The CFSE fluorescence label also showed a layer scan circular structure on the surface of the needle body, wrapped around the outer layer of Rhodamine B fluorescence. The thickness of the CFSE fluorescence layer was about twice that of the control group. The DAPI fluorescence label showed a darker layer scan circular structure at the bottom of the microneedle, and the DAPI fluorescence label was extremely dark or invisible near the needle tip. Rhodamine B equipped with A20 whole-cell vaccine is basically consistent with CFSE fluorescence labeling and A20–ICD group, with CFSE fluorescence layer having a thicker thickness, and DAPI fluorescence labeling showing bright layer scanning circular structures in the overall range of microneedles, and the fluorescence intensity was relatively uniform.

From the fluorescence layer scanning results of PLGA porous microneedles in each group by confocal microscopy (Figure 8), it can be seen that the control group GelMA also shows CFSE fluorescence layer, but the fluorescence layer is thinner, which was speculated to be due to the binding of CFSE dye to free amino groups in incompletely modified gelatin, while DAPI shows no fluorescence at all; The microneedles of each experimental group carrying A20 with ICD showed a thick CFSE fluorescence layer, which was the effect of cell protein and free amino in GelMA hydrogel showing fluorescence together. In the experimental group, the A20–ICD group only showed DAPI fluorescence with layer scanning circular structure at the bottom of the microneedles, and the A20 whole-cell vaccine group showed bright and uniform DAPI fluorescence layer scanning circular structure in the whole range of the microneedles. We used DAPI fluorescence as the decisive indicator for determining the loading of A20 whole-cell vaccine and CFSE fluorescence as the auxiliary indicator. We believed that A20 whole-cell vaccine achieves the most ideal PLGA porous microneedle loading effect, while A20–ICD has poor loading effect.

**Figure 9:**
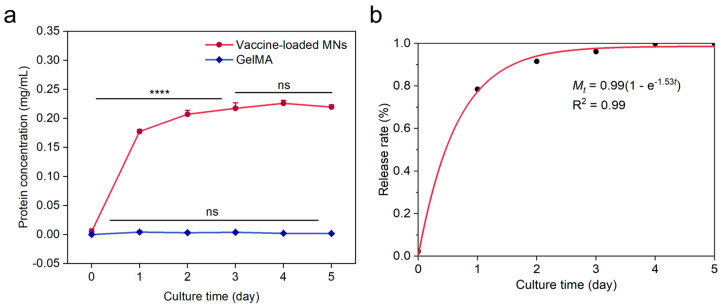
(**a**) Porous microneedle system release of A20 whole-cell vaccine. (**b**) First order equation release model fitting of porous microneedle patch system for whole-cell vaccine. **** *p*  <  0.0001, ns: not significant.

Dispersed A20 whole-cell vaccine in GelMA and mount them onto PLGA porous microneedle patches to obtain a porous microneedle patch system loaded with whole-cell vaccines. BCA kit was used to continuously detect the protein release amount of the microneedle patch system in PBS solution, which was used as an indicator for the release of A20 whole-cell vaccine from the porous microneedle system, and the protein release amount of empty GelMA hydrogel without carrier in PBS solution was used as a control. The results showed (Figure 9a) that within 3 days after being loaded into the microneedle system, the protein content released by the microneedle system in the A20 whole-cell vaccine continued to significantly increase, and tended to stabilize at 3–5 days, with a release rate showing a characteristic of first fast and then slow. GelMA did not detect any protein release that could be indicated by the BCA assay kit, confirming that the presence of GelMA did not affect the release experiment of whole-cell vaccines.

The release or dissolution of drugs is a complex process, and its release pattern can usually be fitted by drug release models based on zero order equations, first-order equations, or Highchi equations. The release pattern of the whole-cell vaccine porous microneedle patch system in this study is similar to a first-order equation release model (defined as $M_{t}$ as the cumulative release amount at time *t*, $M_{\infty }$ as the cumulative release amount at time ∞, and $M_{t}$/$M_{\infty }$ as the cumulative release percentage at time *t*).

$$\ln\left(1-\frac{M_{t}}{M_{\infty }}\right)=-kt$$

Based on this, the release pattern of the porous microneedle patch system for whole-cell vaccines was fitted (Figure 9b), and it can be seen that the fitting result R^2^ > 0.99. It is believed that the release process of whole-cell vaccines conforms to the small molecule first-order equation release model, which is also a common release model for polymer skeleton drug delivery systems. We believe that it has basically achieved the expected release effect. The fitting equation is,

$$M_{t}=0.99\;(1-e^{-1.53t})$$

However, considering the weakened influence of autocatalytic effects in porous PLGA-based materials, their drug release patterns may not fit well with first-order equation release models. Early drug release amounts may be overestimated, while late drug release amounts may be underestimated [57]. Therefore, we need to realize that this equation only showed a preliminary and possible fitting trend of porous microneedles releasing whole-cell vaccines, and its more appropriate drug release fitting law still needs further exploration.

## 3. Conclusions

Current clinical treatment of DLBCL faces challenges such as adverse reactions, poor patient compliance, low cure rate, and high recurrence rate. Recent exploratory studies on lymphoma therapy have shown promising results. For lymphoma with unclear specific antigens, a tumor whole-cell vaccine that covers the entire series of tumor-associated antigens has a special advantage. This vaccine retains the immunogenicity of tumor cells and removes their tumorigenicity, offering the potential for comprehensive and efficient long-term treatment of DLBCL. Based on this background, we designed a method to prepare a malignant lymphoma whole-cell vaccine and verified its immune cell activation effect against malignant lymphoma. Specifically, the photothermal effect mediated by PBNPs induced ICD in A20 cells to produce A20 whole-cell vaccine. We determined the most suitable LPD conditions for photothermal treatment and detecting ICD markers and proved that A20 cells lose tumorigenicity and retain immunogenicity, highlighting the superiority of photothermal treatment over other thermal treatment methods. The obtained A20–ICD was ultrasonic broken to expose more antigens and further enhance immunogenicity. In vitro verification based on mouse primary BMDCs and T cells to verify that the A20 whole-cell vaccine had the immune activation effect. Finally, PLGA porous microneedles with sufficient mechanical strength and mounting space were designed and prepared. GelMA was used as the drug-filling component for the pores of PLGA porous microneedles, achieving the construction and characterization of an intradermal drug delivery system. The loading of A20 whole-cell vaccine in porous microneedle patches has been achieved, and the loading and release effect of A20 whole-cell vaccine has been verified. Its release conforms to the first-order kinetic release process model of small molecule drugs, and the expected loading and release effects have been basically achieved. This tumor whole-cell vaccine combined with porous microneedle system has the potential to realize broad-spectrum immunotherapy against DLBCL and offers a new direction for the development of DLBCL therapy. Considering the practical application of whole-cell vaccines, they may be combined with immune adjuvants in the future to further enhance therapeutic efficacy. In addition, further experimental verification is required to determine the frequency, dosage, and in vivo efficacy of the A20 whole-cell vaccine. The in vivo efficacy of mouse model is still being further validated.

## 4. Materials and Methods

### 4.1. Materials

Tween 20, rhodamine B, and ethanol absolute were purchased from Sinopharm Chemical Reagents Ltd. (Shanghai, China). n-arm-PLGA (Mw 5–15 kDa, lactic: glycolic 75: 25, random, n = 0, 1, 2, 3, or 4) was purchased from Ruixi Ltd. (Xi’an, China). 2-Hydroxy-1-ethanethiol, triethylamine (TEA), methacryloyl chloride, 2,2′-azobisisobutyronitrile (AIBN), CaCO_3_ nanoparticles (particle size ≤ 30 μm), and dimethyl sulfoxide (DMSO) were purchased from Aladdin Biochemical Technology Co., Ltd. (Shanghai, China). Polyethylene glycol diacrylate (PEGDA, Mn 250 Da) was sourced from Merck Co., Ltd. (Shanghai, China). Linear PLGA (lactic: glycolic 75: 25, Mw 76–115 kDa, random) was sourced from Shandong Medical Device Research Institute (Shandong, China). Polydimethylsiloxane (PDMS) microneedle mold was sourced from Weina Benteng Ltd. (Henan, China). Methacryloyl gelatin (GelMA, Mw 100–200 kDa, degree of amino substitution 60 ± 5%) and photoinitiator LAP were sourced from EngineeringForLife Ltd. (Suzhou, China). Fetal bovine serum (FBS) was sourced from Gibco of Thermo Fisher Scientific Inc. (Waltham, MA, USA). Phosphate buffered saline (PBS), Dulbecco’s modified Eagle’s medium (DMEM) high-glucose, Roswell Park Memorial Institute (RPMI) medium 1640, ready-to-use DAPI dye solution, BCA protein assay kit, and were provided by KeyGen Biotechnology Co., Ltd. (Nanjing, China). Mannitol was purchased from Yuanye Bio-Technology Co., Ltd. (Shanghai, China). QuickBlock^TM^ blocking buffer for immunol staining was obtained from Beyotime Biotechnology Co., Ltd. (Shanghai, China). Lipopolysaccharide (LPS) was purchased from Macklin Co., Ltd. (Shanghai, China). Glycine was purchased from Meryer Co., Ltd. (Shanghai, China). Bovine serum albumin (BSA) was purchased from Biofroxx (Einhausen, Germany). Goat anti-rabbit IgG secondary antibody AF488 conjugated and general adenosine triphosphate ELISA kit were purchased from Sabbiotech (Baltimore, MD, USA). Primary antibodies against specific intracellular molecules for immunocytochemistry were ordered from Abcam (Cambridge, UK). Recombinant murine IL-4, Recombinant murine IL-2, Recombinant murine GM-CSF, and RBC lysis buffer were ordered from PeproTech (Rocky Hill, CT, USA). 5-Carboxyfluorescein diacetate N-succinimidyl ester (CFSE) was ordered from MedChemExpress (Monmouth Junction, NJ, USA). Purified anti-mouse CD3ε antibody, purified anti-mouse CD28 antibody, purified anti-mouse CD16/32, FITC anti-mouse CD11c antibody, PE anti-mouse CD80 antibody, APC anti-mouse CD86 antibody, FITC anti-mouse CD3 antibody, PE anti-mouse CD25 antibody, APC anti-mouse CD69 antibody, APC anti-mouse CD3 antibody, and zombie NIR^TM^ fixable viability kit were purchased from BioLegend (San Diego, CA, USA). Prussian blue nanoparticles (PBNPs) were gifted from M. Qian-Qian Li at Southeast University (Nanjing, China) [58]. A20 cells were ordered from Cellcook Co., Ltd. (Guangzhou, China). BALB/c mice (9 weeks, female) were obtained from Jiangsu Qinglongshan Biotechnology Co., Ltd. (Nanjing, China). Unless otherwise stated, none of the above reagents or materials have undergone further processing.

### 4.2. Cell Culture

A20 and mouse primary bone marrow cells were cultured in RPMI-1640 medium (KeyGen, China) containing 1% streptomycin/penicillin, 0.05 mM 2-mercaptoethanol, and 10% FBS (Gibco, Life technologies, Grand Island, NY, USA). Cells were cultured at 37 °C in a humidified atmosphere with 5% CO_2_.

### 4.3. Photothermal Effect of PBNPs Experiments In Vitro

A20 cells were seeded onto 96-well culture plates at a density of 1 × 10^6^ cells/mL and were incubated with PBNPs (175.8 nm) at a density of 0.1 mg/mL for 24 h. After 24 h, the culture medium was irradiated by an 808 nm laser (PURI Materials, Shenzhen, China) with LPDs of 0.25 W/cm^2^, 0.5 W/cm^2^, 0.75 W/cm^2^, and 1 W/cm^2^, respectively for 15 min. The whole trace of solution temperature was recorded by a thermocouple thermometer (TASI Electronics, Suzhou, China).

### 4.4. Evaluation of Photothermal Effect Mediated by PBNPs on Cell Viability

A20 cells (1 × 10^6^ cells/mL) were seeded in 96-well culture plates with PBNPs (0.1 mg/mL) 24 h before the experiment. Subsequently, the cells were irradiated by an 808 nm laser with LPDs of 0.25 W/cm^2^, 0.5 W/cm^2^, 0.75 W/cm^2^, and 1 W/cm^2^, respectively for 15 min. After the laser treatment, the cells were gently washed with PBS, and calcein AM/PI staining working solution was added to incubate with the cells for 30 min at room temperature in the dark. Cells were washed with PBS again and measured with flow cytometer (Thermo Fisher Scientific, USA).

### 4.5. Evaluation of the ICD Markers’ Expression

The three major hallmarks of ICD—the exposure of calreticulin (CRT) on the cell surface, the release of adenosine triphosphate (ATP) and high-mobility group box 1 (HMBG1) into the extracellular space—were selected and characterized. A20 cells (1 × 10^6^ cells/mL) were seeded in 96-well culture plates with PBNPs (0.1 mg/mL) 24 h before the irradiation by an 808 nm laser with LPDs of 0.5 W/cm^2^, 0.75 W/cm^2^, and 1 W/cm^2^, respectively for 15 min. The cells were then incubated continuously in cell culture incubators for 4 h to induce ICD, which was followed by centrifugation, the supernatant and the cells were collected respectively.

General ATP concentrations in cell culture supernates of each LPD were then quantitatively determined by general ATP ELISA kit (SABbiotech, USA). Briefly, 50 μL of the supernatant samples or the prepared standard solution, the detection working solution, substrate solution, and stop solution were added into each well of the assay plate successively, and the samples were tested with a microplate reader set to 450 nm.

The expression levels of CRT on the cell membrane surface and HMGB1 in the cytoplasm were characterized by the immunofluorescence method. The cells were fixed with 4% paraformaldehyde solution, blocked with blocking buffer, and incubated overnight with anti-CRT antibody or anti-HMGB1 antibody. After primary antibody incubation, the cells were incubated with AlexaFluor 488-secondary antibody for 1 h, then tested by flow cytometry, or treated with DAPI staining solution and tested with laser scanning confocal microscopy (TCS SP8, Leica Microsystems, Leica, Heidelberg, Germany). Cells were washed with PBS after each staining incubation step.

### 4.6. In Vitro PBNPs Effectively Induced ICD

To confirm the ability of PBNPs to induce ICD, the expression of ICD markers caused by PBNPs was compared with that caused by ordinary water bath heat treatment. For the photothermal treatment of PBNPs group, A20 cells (1 × 10^6^ cells/mL) were seeded in 96-well culture plates with PBNPs (0.1 mg/mL) 24 h before the irradiation by an 808 nm laser with LPD of 0.75 W/cm^2^, respectively for 15 min. For the water bath heat treatment group, A20 cells (1 × 10^6^ cells/mL) were heated in a 60 °C water bath for 13 min. Both groups of cells were then incubated continuously in cell culture incubator for 4 h to induce ICD. Consistent with the above method, ATP concentrations in cell culture supernates were quantitatively determined by general ATP ELISA kit, and the expression levels of CRT on the cell membrane surface and HMGB1 in the cytoplasm were characterized by the immunofluorescence method.

### 4.7. Preparation of A20 Whole-Cell Vaccine by Ultrasonication

A20 cells (1 × 10^6^ cells/mL) were seeded in 96-well culture plates with PBNPs (0.1 mg/mL) 24 h before an 808 nm laser irradiation with a LPD of 0.75 W/cm^2^ respectively for 15 min. The cells were then centrifuged to collect cell pellets, which were resuspended in PBS to obtain A20–ICD. The cell solutions were disrupted with an ultrasonic cell pulverizer (20–25 kHz, SCIENTZ, Ningbo, China) at powers of 162.5 W (25%), 325.0 W (50%), 422.5 W (65%), and 487.5 W (75%), respectively (100%: 650 W). The cell solutions were placed in an ice bath throughout the process, and the ultrasound was set to turn on for 2 s and off for 10 s. The cycle worked for 10 min, and the actual ultrasound duration was 100 s. Set the control group to 0.1 mg/mL PBNPs. The particle sizes of A20 cell lysates were measured with Mastersizer (Malvern, UK), and the particle morphology of A20 cell lysates was characterized with the TEM (FEI Tecnai G2 20, FEI Co., Hillsboro, OR, USA).

### 4.8. Generation of Primary T Cells and BMDCs

Primary T cells and BMDCs were collected from the spleen and femurs of 9-week-old BALB/c female mice, respectively. The collected cells were treated with red blood cell (RBC) lysis buffer to deplete RBC, and the cell suspension was sequentially passed through 70 μm and 40 μm cell filters. After that, spleen cells were differentiated in RPMI 1640 complete medium supplemented with 50 ng/mL IL-2, 1 μg/mL anti-mouse-CD3, and 1 μg/mL anti-mouse-CD28 for T cells, bone marrow cells were differentiated in RPMI 1640 complete medium supplemented with 50 ng/mL IL-4 and 50 ng/mL GM-CSF for BMDCs. The culture medium was replaced every two days. On the sixth day, the cells were collected for subsequent experiments.

### 4.9. In Vitro DCs and T Cells Activation by A20 Whole-Cell Vaccine

A20–ICD and A20 whole-cell vaccine were prepared as described above. For BMDCs maturation experiments, the collected BMDCs were resuspended at 1 × 10^6^ cells/mL in complete culture medium with A20–ICD, or A20 whole-cell vaccine, or 1 μg/mL LPS, or 75% ethanol. After 48 h of culture cells were harvested and analyzed.

For T cell maturation experiments, the collected BMDCs were resuspended at 1 × 10^6^ cells/mL in complete culture medium with BMDCs stimulated by A20–ICD, A20 whole-cell vaccine, 1 μg/mL LPS, or 75% ethanol. After 24 h of culture, cells were harvested and analyzed.

### 4.10. Preparation of PLGA Porous Microneedles

We referred to the previously reported method and made adjustments and improvements to prepare PLGA porous microneedles (Figures S1–S3) [59]. Specifically, the n-arm-PLGA (5.0 g) was dissolved in dichloromethane (20 mL) mixed with TEA (1.0 g), and placed into an ice bath. The methacryloyl chloride (1.0 g) was dissolved in dichloromethane (6 mL) and added to the PLGA solution by drops. Eight hours later, the product was purified by dialysis in DMF (10 mL product in 200 mL DMF) for another two days. The final product (n-arm-PLGA-Acry) was obtained by precipitation in water as a white solid. The n-arm-PLGA-Acry was dissolved in dioxane with a final concentration of 500 mg/mL, azodiisobutyronitrile (AIBN) was dissolved in Dioxane with a concentration of 100 mg/mL and the linear PLGA was dissolved in Dioxane with a concentration of 200 mg/mL. Then, 300 mg of n-arm-PLGA-Acry, 150 mg of triethylene glycol diacrylate (TEGDA), 10 mg of AIBN, 20 mg of PLGA and 90 mg of CaCO_3_ microparticles were mixed and added to a polydimethylsiloxane (PDMS) micromold (needle length 700 μm, number of microneedles 15 × 15, needle base diameter 340 μm, needle tip diameter 15 μm) with dioxane pre-filled into the needle. Four hours later, the microneedle was cross-linked overnight at 90 °C before the microneedle patch was peeled off. The microneedle patch was placed in HCl/dioxane solution for 2 h for swelling, and then water was added to initiate the reaction between HCl and CaCO_3_, accompanied by CO_2_ bubbles generated. Finally, the Porous Microneedles patch was treated with plasma to generate a hydrophilic surface. The synthesis and crosslinking effects of the material were characterized by nuclear magnetic resonance hydrogen spectroscopy and mass spectrometry. The morphology of the Porous Microneedles patch was characterized by SEM (ZEISS Ultra Plus, Carl Zeiss AG, Oberkochen, Germany) and statistically analyzed using ImageJ software 1.5.3 version.

### 4.11. Loading and Release of Whole-Cell Vaccines

Dispersed the whole-cell vaccine in a 5% GelMA solution and add a photoinitiator LAP. Evenly dispersed A20–ICD and A20 whole-cell vaccine in GelMA solution, and immersed PLGA porous microneedle patch in the solution. Vacuum at 60 °C for 2 h, then inverted at 60 °C under normal pressure for 30 min to eliminate excess solution. Subsequently, the porous microneedle system was irradiated with a light power density of 6 W/cm^2^ at a wavelength of 405 nm blue light for 10 s for photocuring. The PLGA microneedle was labeled with Rhodamine B, and the protein and nucleic acid were labeled with CFSE and DAPI staining solutions, respectively. The fluorescence results of both methods comprehensively showed the loading status of the A20 whole-cell vaccine. The results were characterized using a fluorescence confocal microscope. Soak the porous microneedle system loaded with whole-cell vaccines in PBS, seal the system, incubate at 37 °C, and measure BCA protein content every 24 h to characterize drug release.

### 4.12. Data Statistical Analysis

Statistical analysis was performed using Origin software 10.1 version. Tukey test was used to compare three or more groups. T-test was used to compare two groups. *p*-values < 0.05 were considered statistically significant. Data are presented as means ±  standard error of the mean. * *p*  <  0.05, ** *p*  <  0.01, *** *p*  <  0.001, **** *p*  <  0.0001, ns: not significant.

## Data Availability

The original contributions presented in this study are included in the article/Supplementary Material. Further inquiries can be directed to the corresponding author.

## Supplementary Materials

The following supporting information can be downloaded at: https://www.mdpi.com/article/10.3390/gels10120838/s1, Figure S1. (a) Synthesis principle of n-arm-PLGA. (b) The synthesis principle of n-arm-PLGA-Acry. (c) The synthetic route of n-arm-PLGA-Acry; Figure S2. (a) Principle of PLGA prepolymer liquid thermal crosslinking. (b) GelMA photopolymerization principle; Figure S3. (a) n-arm-PLGA-Acry 1H NMR and sample photos. (b) Material spectra of n-arm-PLGA, n-arm-PLGA-Acry, and PLGA pre polymerized liquid thermal interaction products; Figure S4. Puncture ability of PLGA porous microneedle patch (a) Material testing machine compression fracture curve. (b) H&E stained tissue sections of mouse skin tissue puncture model. (b) Fluorescence (Rhodamine B) image of the dermal needle tip in a mouse skin tissue puncture model under confocal microscopy; Figure S5. (a) GelMA hydrogel was obtained by UV curing of GelMA prepolymer solution, and further cultivated in the culture medium. The image of morphological characteristics changes occurred in this process. (b) Rheological properties of GelMA prepolymer and GelMA hydrogel. (c) FTIR of GelMA prepolymer solution and GelMA hydrogel. (d) Swelling rate change curve and morphological characteristics change image of GelMA hydrogel in acellular protein-free simulated body fluid (SBF); Figure S6. (a) GelMA hydrogel 3D uniformly carries cells. (b) Cytotoxicity of GelMA hydrogel.
