# Supplementary material for: Whole-Cell Vaccine Preparation Through Prussian Blue Nanoparticles-Elicited Immunogenic Cell Death and Loading in Gel Microneedles Patches
Source: Gels. 2024 Dec 19;10(12):838. doi: 10.3390/gels10120838 (PMC11675167; doi:10.3390/gels10120838)
Supplement: Supplementary file 1 [file gels-10-00838-s001.zip › gels-3371204-supplementary.pdf]

Supplementary Data for

Whole-cell Vaccine Preparation through Prussian Blue Nanoparticles-Elicited Immunogenic Cell Death and Loading in Gel Microneedles Patches

Wen-Xin Fu[1], Qian-Qian Li[1], Jingyi Sheng[2], Haoan Wu[1], Ming Ma[1], Yu Zhang[1, *]

[1] Jiangsu Key Laboratory for Biomaterials and Devices, School of Biological Science and Medical Engineering & Basic Medicine Research and Innovation Center of Ministry of Education, Southeast University, Nanjing 211102, P. R. China

[2] Jiangsu Key Laboratory for Biomaterials and Devices, School of Biological Science and Medical Engineering, Southeast University, Nanjing 210096, P. R. China

* Correspondence: zhangyu@seu.edu.cn

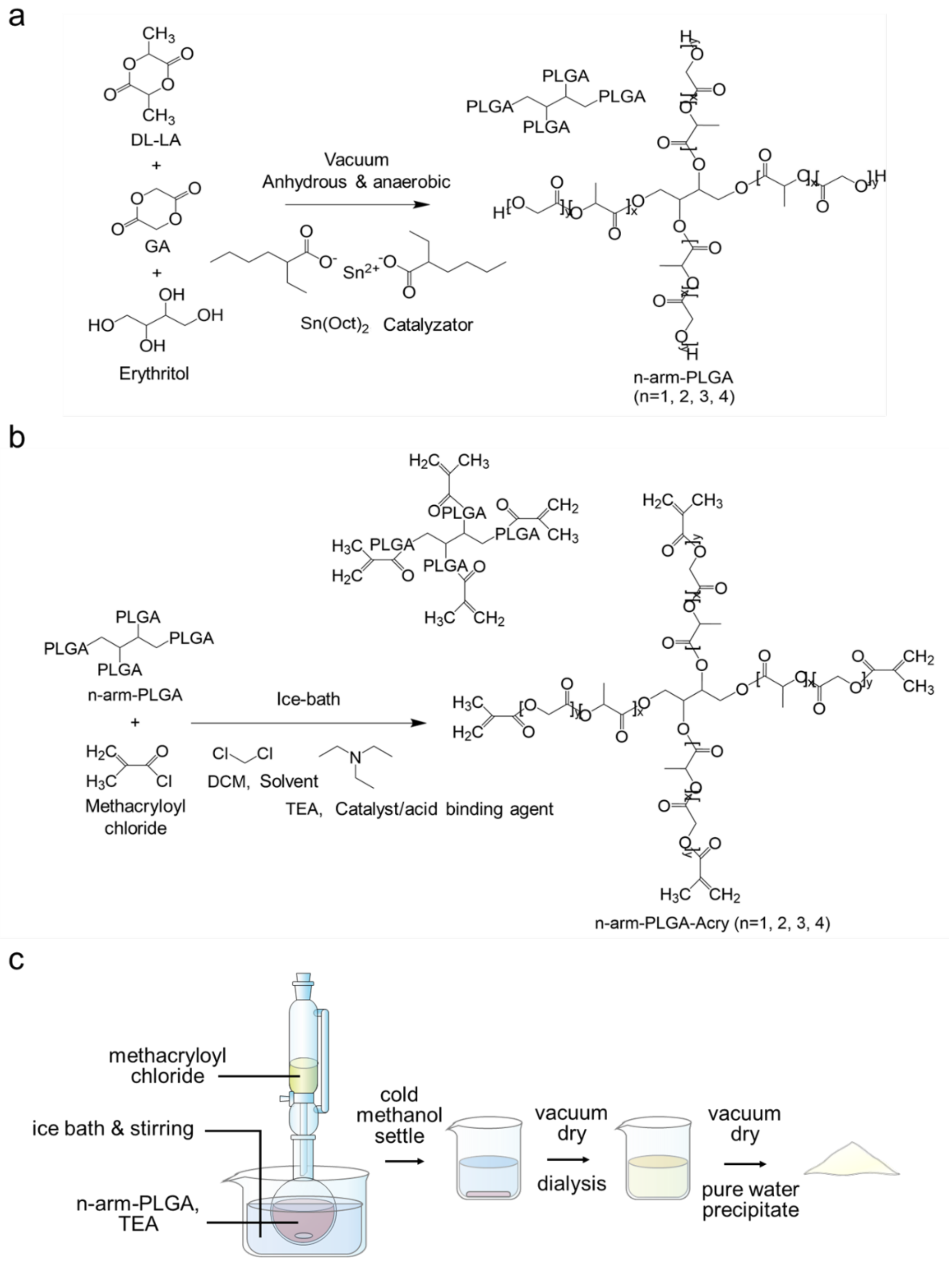

Figure S1. (a) Synthesis principle of n-arm-PLGA. (b) The synthesis principle of n-arm-PLGA-Acry. (c) The synthetic route of n-arm-PLGA-Acry.

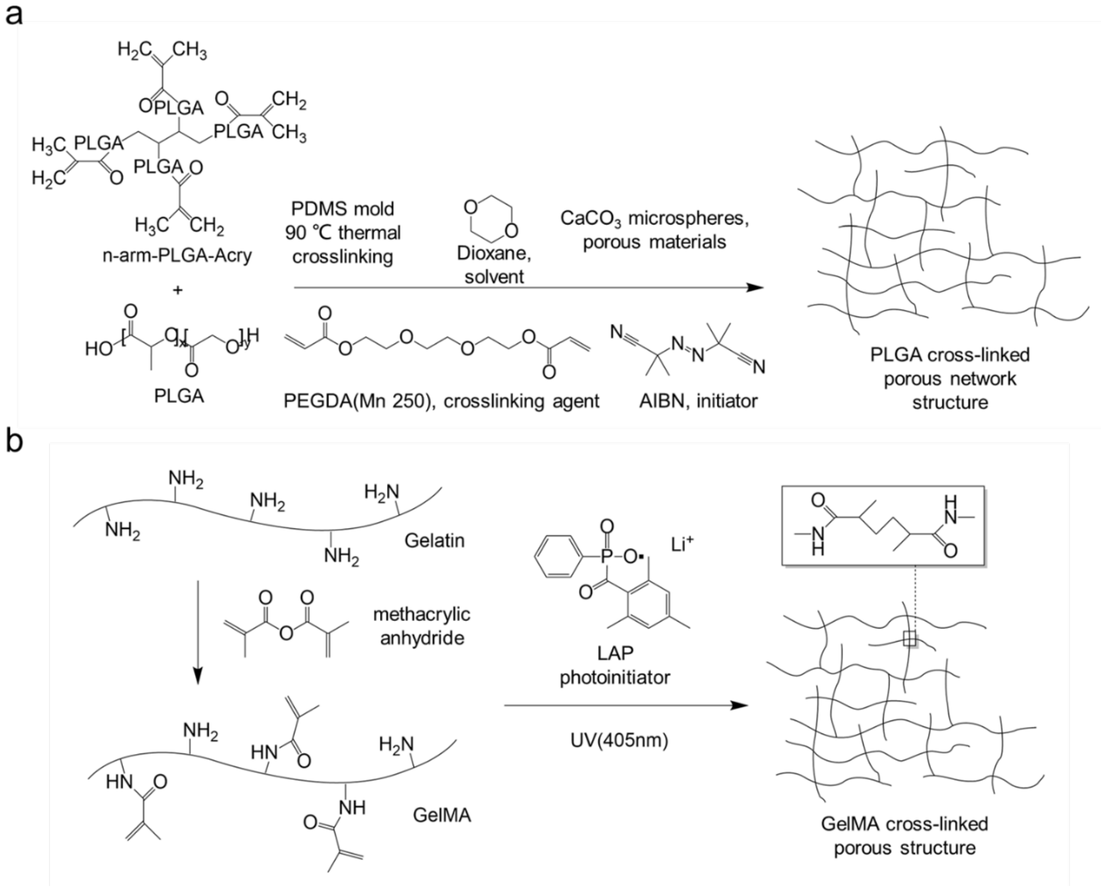

Figure S2. (a) Principle of PLGA prepolymer liquid thermal crosslinking. (b) GelMA photopolymerization principle.

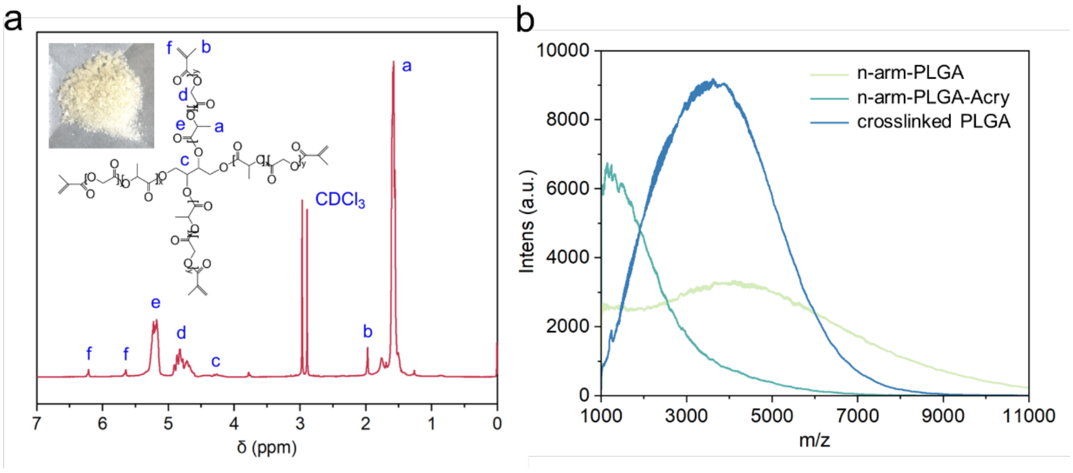

Figure S3. (a) n-arm-PLGA-Acry ^{1}H NMR and sample photos. (b) Material spectra of n-arm-PLGA, n-arm-PLGA-Acry, and PLGA pre polymerized liquid thermal interaction products.

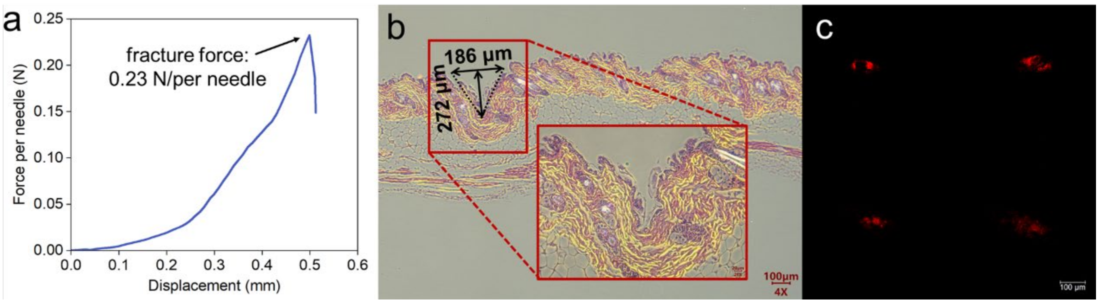

Figure S4. Puncture ability of PLGA porous microneedle patch (a) Material testing machine compression fracture curve. (b) H&E stained tissue sections of mouse skin tissue puncture model. (c) Fluorescence (Rhodamine B) image of the dermal needle tip in a mouse skin tissue puncture model under confocal microscopy.

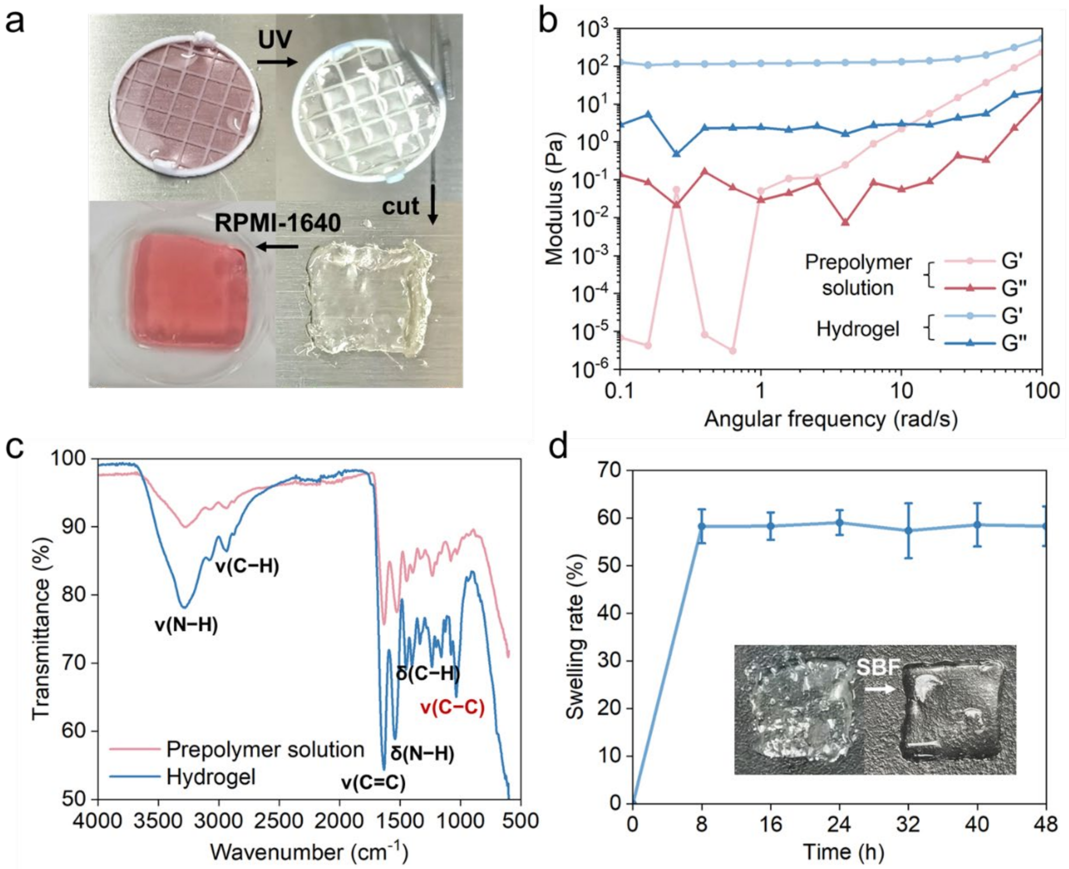

Figure S5. (a) GelMA hydrogel was obtained by UV curing of GelMA prepolymer solution, and further cultivated in the culture medium. The image of morphological characteristics changes occurred in this process. (b) Rheological properties of GelMA prepolymer and GelMA hydrogel. (c) FTIR of GelMA prepolymer solution and GelMA hydrogel. (d) Swelling rate change curve and morphological characteristics change image of GelMA hydrogel in acellular protein-free simulated body fluid (SBF).

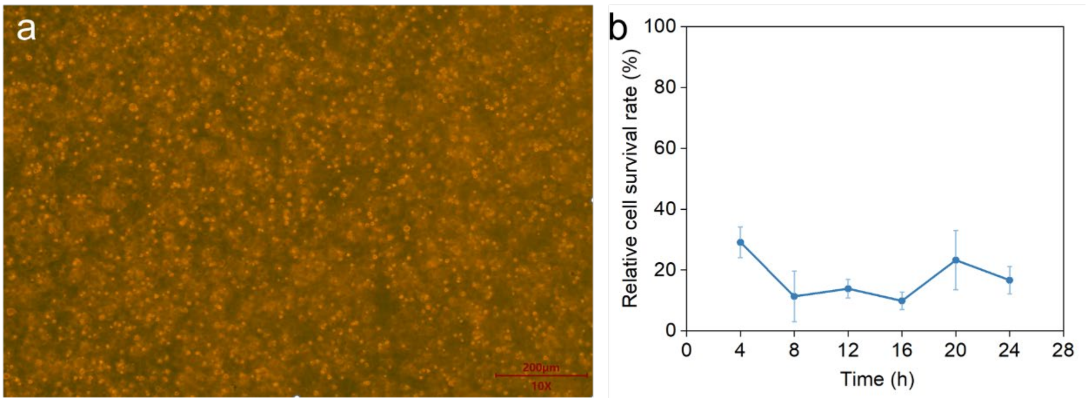

Figure S6. (a) GelMA hydrogel 3D uniformly carries cells. (b) Cytotoxicity of GelMA hydrogel.
